# A Child without Calvaria or Brain

**Published:** 1869-01-01

**Authors:** L. Redmond

**Affiliations:** Preston, Minn.


### A CHILD WITHOUT CALVARIA OR BRAIN.

Reported by L. Redmond, M.D., Preston, Minn.

Editor of Chicago Medical Journal:

On the evening of the 14th of November last, I was called to see Mrs. D. On my arrival I was informed that she had given birth to a still-born female child, which, on examination, I found to be perfect in form, with the following exception, viz.: There was not the slightest trace of a calvaria or brain. Both parietal, the occipital and frontal bones were entirely absent, excepting the orbital plates of the latter. Stretching across the uneven surface of the base of the skull, was a thin transparent membrane, which seemed to be a continuation of the skin. Beneath this membrane there was a small quantity of serous fluid of an amber color. On making inquiry, I was told, by Mrs. D., that during her gestation she had enjoyed good health, up to within six or eight weeks of her confinement, when, on returning from the spring one day with a pail of water, she slipped and fell with her right side on the pail. Since the above unfortunate accident she has been very miserable, part of the time up and about, the remainder in bed, but suffering more or less pain all the time throughout the abdomen, but especially in the right side. I should state in this connection, that since the fall Mrs. D. is not conscious of having felt any movement of the child whatever.

Now, Mr. Editor, what was the cause of this malformation? Did the fall upon the pail injure the child's head, so that inflammation and softening of the brain (or rather its liquefaction, including the contiguous osseous structures) was the result? or, are we to look to some obscure and inexplicable cause for this remarkable malformation?

